# A Great American Hospital

**Published:** 1889-05-11

**Authors:** 


### A Great American Hospital.

On our table lies an invitation to be present at the opening of the Johns Hopkins Hospital, at Baltimore, on the 7th May. We could not avail ourselves of it, but it sets one thinking of an institution which has strong claims to be considered the finest in the world, and of its founder—a man who, one would have thought from his life, had no special interest in either education or charity.

Johns Hopkins—his name was Johns, not, as many people might fancy, John—was the son of a poor Quaker, and came to Baltimore to serve as clerk in a store kept by his uncle, who was a merchant of some standing. There he showed himself so shrewd and industrious, that he soon had entire control of the establishment. Then his uncle set him up in business for himself, the result of which was that in a few years he was able to buy out his uncle. After that everything he touched seemed to turn into gold. Money-making became his ruling passion. His one idea was accumulation. To his natural shrewdness he added parsimony. He never spent a cent more than he could help. Even after he had millions to his credit he would argue for a half hour, if necessary, to get a reduction on the smallest purchase. He and John W. Garrett were the financial autocrats of the city. He profited largely from investments in Baltimore and Ohio stock, and when he died he was many times a millionaire.

The bulk of this fortune went to the establishment of a hospital and a university to bear his name. The university was opened in 1876, and its growth since then has been without a parallel in the history of education. The number of students has increased from 89 the first year to about 400 in 1888. The number of teachers has increased from 20 in 1876-7 to about 50 at present. Every State in the Union and nearly all foreign countries are represented. Most of the famous men of science and letters have lectured there, and the institution is having a very perceptible influence upon the social life of the city, and is giving it an intellectual phase which it never possessed before.

Johns Hopkins was even more interested in his hospital than in the university, as is proved by the fact that his bequest for it was much the larger. His idea was that it should be absolutely the finest hospital in the world, and that it should be entirely free for all classes and conditions of humanity. In 1875 the trustees decided to get the views of five of the most distinguished physicians, chosen from different parts of the country, who had made hospitals their special study. One of these, Dr. Billings, surgeon in the United States army, and no mean authority on hospitals, was invited to act as medical superintendent and director of the buildings which were in course of erection. He was sent to Europe to visit all the noted hospitals in England and on the Continent.

The result is one of the most excellent collections of buildings in the world. They are twenty-three in number, and as they stand on three of the most elevated squares in Baltimore, being 115 feet above tide-water, they are conspicuous from every outlook in the city. From the main building, whose dome rises 200 feet above the ground and 315 feet above tidewater, there is a superb view of all Baltimore and its surroundings.

The general shape of the buildings is that of an E. The administration building, the largest and imposing of all, stands in the front centre of the group. It contains the offices of the trustees and the faculty and several lecture-halls. Extending from it to and through all other structures is a wide and lofty corridor. The top of this corridor forms an open terrace walk on a level with the hospital ward floors in the second storeys of the several buildings.

The wards extend eastward from the administration building in two lines. The first two wards are octagon, and will be for paying patients. The others are the common wards. In the main department of each ward will be twenty-four beds. There are two isolation wards, each with its separate system of ventilation and other facilities, so that they can be completely shut off from the rest of the hospital. In the administration building, besides the offices and library, are the private apartments for the resident medical officers and students. The pay wards are in the octagonal building, and contain private bathrooms, dining-saloons, and kitchens. The wards contain over 300 beds.

The next principal building to the administration building is the nurses' home, which is four storeys in height. The two upper storeys are apportioned into sleeping apartments, each nurse having her own room. On the lower floors are reception-rooms and reading-rooms exclusively for the nurses, and a training kitchen. This will be used for giving the student nurses thorough training in the preparation of food for invalids. A feature of the institution is to be a training school for nurses, who will be given careful medical instruction and practice for a period of several years in the wards of this hospital before receiving their certificates.

The laundry is about as large as an ordinary hospital. It is one of the most interesting of the buildings, the special feature being its complete disinfecting chamber. The bath-house is supplied with the latest inventions for all kinds of baths. There is also a dispensary for the treatment of the poor of the city. It is a commodious building, containing a central waiting-room, twelve private consulting-rooms, and rooms specially adapted to surgical operations.

The serious questions of drainage, ventilation, and heating have received due attention, and in the matter of sanitation the hospital promises to be as nearly perfect as may be. The heating will be done by hot-water pipes, and the lighting by electricity. There will be a medical school in connection with the hospital, and the educational buildings are worthy of the rest of the establishment. Although not, as enthusiastic natives of Baltimore declare, the largest hospital in the world—it is smaller than eight in the British Isles alone, not to speak of the immense hospitals at Milan and elsewhere, the Johns Hopkins Hospital is undoubtedly one of the most complete and perfect. With Dr. Billings at its head, who we cordially congratulate on the successful completion of an arduous and difficult task, there can be no doubt that it will be admirably managed, and in all respects worthy of imitation.

